# Hypoxia, oxidative stress, and the interplay of HIFs and NRF2 signaling in cancer

**DOI:** 10.1038/s12276-024-01180-8

**Published:** 2024-03-01

**Authors:** Taegeun Bae, Steffanus Pranoto Hallis, Mi-Kyoung Kwak

**Affiliations:** 1 Integrated Research Institute for Pharmaceutical Sciences, The Catholic University of Korea, Bucheon, Gyeonggi‑do 14662 Republic of Korea; 2 Department of Pharmacy, Graduate School of The Catholic University of Korea, Bucheon, Gyeonggi‑do 14662 Republic of Korea; 3 College of Pharmacy, The Catholic University of Korea, Bucheon, Gyeonggi‑do 14662 Republic of Korea

**Keywords:** Cancer therapeutic resistance, Cancer therapeutic resistance

## Abstract

Oxygen is crucial for life and acts as the final electron acceptor in mitochondrial energy production. Cells adapt to varying oxygen levels through intricate response systems. Hypoxia-inducible factors (HIFs), including HIF-1α and HIF-2α, orchestrate the cellular hypoxic response, activating genes to increase the oxygen supply and reduce expenditure. Under conditions of excess oxygen and resulting oxidative stress, nuclear factor erythroid 2-related factor 2 (NRF2) activates hundreds of genes for oxidant removal and adaptive cell survival. Hypoxia and oxidative stress are core hallmarks of solid tumors and activated HIFs and NRF2 play pivotal roles in tumor growth and progression. The complex interplay between hypoxia and oxidative stress within the tumor microenvironment adds another layer of intricacy to the HIF and NRF2 signaling systems. This review aimed to elucidate the dynamic changes and functions of the HIF and NRF2 signaling pathways in response to conditions of hypoxia and oxidative stress, emphasizing their implications within the tumor milieu. Additionally, this review explored the elaborate interplay between HIFs and NRF2, providing insights into the significance of these interactions for the development of novel cancer treatment strategies.

## Hypoxia and oxidative stress: pivotal players in tumor progression

In our daily lives, oxygen is vital for survival, but its levels can vary due to environmental shifts or within our bodies, such as in diseases like heart disease or cancer. However, excess oxygen can also be detrimental, leading to a condition called oxidative stress. Cells have evolved systems to adapt to these fluctuating oxygen levels, with key roles played by proteins named hypoxia-inducible factors (HIFs) and nuclear factor erythroid 2-related factor 2 (NRF2). These factors aid cells’ survival by activating different and overlapping genes that can enhance oxygen supply or shield against damage. This study discovered that HIFs and NRF2 can occasionally collaborate to aid cancer cells’ growth and treatment resistance. The key discoveries suggest that targeting these pathways could be a novel approach to cancer treatment, especially in tumors that have adapted to low oxygen conditions.

This summary was initially drafted using artificial intelligence, then revised and fact-checked by the author.

## Introduction

Oxygen (O_2_) is the most fundamental element for most living organisms on Earth and serves as the final electron acceptor in the mitochondrial energy production process^1^. The concentration of O_2_ encountered by cells undergoes significant changes due to environmental perturbations and the transport and distribution processes within living organisms. Hypoxic conditions, characterized by an insufficient O_2_ supply relative to the required amount, are prevalent in various pathological conditions, such as chronic obstructive pulmonary disease and ischemic heart disease. These conditions are particularly noticeable in the tumor microenvironment (TME), where vasculature is limited^2^. However, although O_2_ is a relatively stable molecule, O_2_-derived free radical formation is proportional to the O_2_ concentration. Inhalation of O_2_ concentrations higher than the normal partial pressure can lead to high reactive oxygen species (ROS) levels. Under these conditions, cells experience oxidative stress, where oxidant production significantly outweighs that of antioxidants and the capacity to repair damaged cellular components by oxidants^3^. Accordingly, cells have evolved sophisticated response systems to acclimatize to variable O_2_ availability. The primary effectors of the cellular hypoxic response are hypoxia-inducible factors (HIFs), including HIF-1α and HIF-2α. These transcription factors activate the expression of an array of genes involved in increasing O_2_ supply (genes regulating erythropoiesis and angiogenesis) and decreasing O_2_ expenditure (genes for O_2_-independent glycolytic metabolism), eventually triggering a cellular adaptive response to an O_2_-restricted environment^2,4^. The opposite of hypoxia, known as hyperoxia, is also a critical condition that induces cellular macromolecule dysfunction and, ultimately, cell death. In response, the expression levels of genes involved in hyperoxia-derived ROS removal and damaged cell repair increase. Nuclear factor erythroid 2-related factor 2 (NRF2) is the primary transcription factor mediating this response and promotes the adaptation and survival of cells under oxidative stress conditions^5^.

Hypoxia and oxidative stress are core hallmarks of solid tumors^6^. During the rapid tumor growth process, insufficient angiogenesis creates a hypoxic environment within the tumor tissue. Indeed, the average pO_2_ is reportedly 10 mmHg (1.4% O_2_) for breast, head, neck, and cervical cancers^7^. Under these conditions, HIFs are activated and promote the expression of various genes that lead to tumor vascularization, epithelial–mesenchymal transition (EMT), invasion/metastasis, cancer-specific metabolism, immune escape, and cancer stem cell (CSC) trait acquisition. Consistent with this, HIF levels in tumor biopsy samples have been associated with high mortality rates in patients with cancer; therefore, chemical HIF inhibitors are under investigation as a therapeutic option for cancer treatment^2^. Compared with noncancer cells, cancer cells exhibit high ROS levels due to oncogene activation, a high metabolic rate, and hypoxic stress^8^. To cope with oxidative stress, cancer cells aberrantly activate NRF2, promoting the expression of genes involved in ROS removal, cell proliferation, apoptotic resistance, and metabolic reprogramming, which ultimately favors cancer growth and progression^9^. In addition, hypoxia and oxidative stress in the TME are connected factors rather than independent factors; therefore, their effects on the HIF and NRF2 signaling systems are notably complex. For instance, hypoxia and oxidative stress often coexist within the TME. Hypoxia can lead to temporally increased ROS production through mitochondrial complex III dysfunction^10^. Notably, oxidative stress also serves as a signaling component of the hypoxic response. ROS can stabilize HIF-1α by inhibiting prolyl hydroxylase domain protein (PHD) activity^11^. Moreover, when hypoxic stress is sustained, mitochondrial ROS generation is reduced through a mechanism involving the substitution of the cytochrome c oxidase subunit and subsequent improvement in electron transfer efficiency^12^. Given the pivotal role of HIFs and NRF2 signaling in the context of tumor growth and progression, gaining a comprehensive understanding of how these systems respond to unique environmental stresses and alterations within the TME is imperative. This review aimed to elucidate the dynamic changes and functions of the HIF and NRF2 signaling pathways, particularly in response to hypoxia and oxidative stress conditions, with a specific emphasis on their implications within the tumor milieu. Furthermore, this review examines the intricate interplay between HIFs and NRF2 in the context of cancer.

## Adaptive response systems to variable O_2_ tensions

### Low O_2_ tension and HIFs

The discovery of HIFs and their regulation by the von Hippel–Lindau tumor suppressor protein (pVHL) and PHDs represents a significant breakthrough in our understanding of cellular responses to hypoxia. HIFs are heterodimeric transcription factor proteins composed of two subunits of the basic helix–loop–helix-Per–Arnt–Sim family: the O_2_-sensitive α subunit and the constitutively active β subunit^13,14^. Among the α subunits, HIF-1α (encoded by *HIF1A*) and HIF-2α (encoded by *EPAS1*) are well-characterized proteins that govern the transcriptional activity of numerous genes and contribute to adaptive strategies in low-O_2_ environments. In contrast, HIF-3α (encoded by *HIF3A*) has been relatively understudied^13^. The HIF-1β subunit (encoded by aryl hydrocarbon nuclear translocator; *ARNT*) heterodimerizes with HIF-α, stabilizing the HIF complex and enabling HIF target gene transcription^14^.

**Fig. 1:**
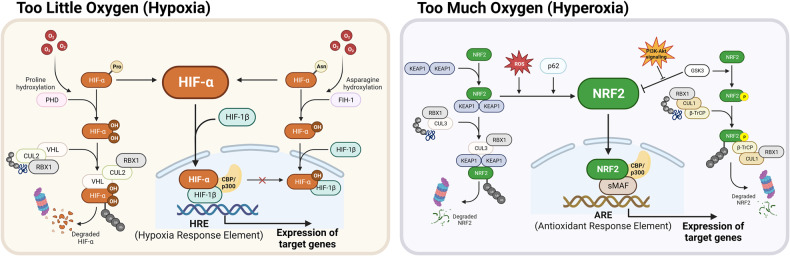
HIFs and NRF2, adaptive response systems to variable oxygen availability. [Left] In hypoxic conditions, HIFs stabilize, translocate into the nucleus, and bind to the HRE, thereby inducing the expression of their target genes. In the presence of oxygen, HIFs are continuously degraded by the canonical PHD/pVHL-mediated pathway. The regulatory mechanism of FIH-1 also contributes to the inhibition of HIF activity. [Right] NRF2 maintains cellular redox homeostasis in response to oxidative stress, which can be induced by an excess oxygen environment. ROS/electrophiles and nonelectrophiles, such as p62, can inhibit NRF2-KEAP1 interactions to activate NRF2 target gene expression. This inhibition occurs through the KEAP1 Cys modification and competition with KEAP1 for binding to NRF2. Additionally, as a KEAP1-independent regulatory pathway, NRF2 phosphorylation by GSK-3β leads to β-TrCP-mediated proteasomal degradation of NRF2.

O_2_ plays an inevitable role in the regulation of HIF-α protein stability. Under normal O_2_ concentrations, O_2_ atoms are inserted into specific proline residues of HIFs (P402/P564 of HIF-1α and P405/P531 of HIF-2α) by PHDs. These hydroxylated HIF-α subunits are subsequently selectively recognized and ubiquitylated by the pVHL-elongin BC-Cullin 2 (CUL2) complex, which induces proteasomal degradation (Fig. 1, left panel)^15^. Notably, mammals possess three characterized PHDs, namely, PHD1–3, which catalyze the hydroxylation of HIF-α subunits in the presence of Fe^2+^ and α-ketoglutarate^16^. These PHDs exhibit varying affinities for HIF-α subunits, with PHD2 primarily targeting HIF-1α, while PHD3 displays greater activity toward HIF-2α than toward HIF-1α^17^. Under hypoxic conditions (0.5–2% O_2_), PHD-driven hydroxylation is inhibited due to a lack of O_2_ as a substrate, resulting in HIF-α accumulation. The translocation of stabilized HIF-α subunits into the nucleus facilitates their dimerization with HIF-1β, leading to the formation of a complex capable of recognizing and binding to the hypoxia response element (HRE; 5′-(A/G)CGTG-3′) in the promoter regions of target genes^13^. In addition to the canonical PHD/pVHL-mediated pathway, another regulatory mechanism involving factor-inhibiting HIF-1 (FIH-1) exists. FIH-1 modulates HIF activity by hydroxylating the asparagine 803 residues within the HIF-α subunits, thereby inhibiting the interaction of HIF-1 with transcription coactivators, i.e., CREB-binding protein and p300 (Fig. 1, left panel)^18^.

Although HIF-1α and HIF-2α share structural similarities and the ability to bind to the same HRE, they exhibit distinct functional characteristics. HIF-1α is ubiquitously expressed in hypoxic tissues, whereas HIF-2α is more selectively expressed in specific tissues, such as the vascular endothelium^19^. HIF-1α is rapidly activated in response to acute hypoxia, leading to the upregulation of genes associated with glycolytic metabolic shift and cell cycle arrest. In contrast, HIF-2α gradually becomes activated under persistent hypoxic conditions, elevating the expression levels of genes involved in erythropoiesis and tumor stemness^19–21^.

HIFs have been shown to upregulate the expression of numerous genes in response to low O_2_ concentrations. Microarray analysis of human pulmonary endothelial cells revealed the upregulation of 245 genes, including those encoding growth factors, cytokines, receptors, signal transduction molecules, and transcription factors, as commonly elevated genes in response to both HIF-1α overexpression and hypoxic incubation^22^. The HIF-1α-regulated genes included vascular endothelial growth factor (VEGF), glucose transporters (GLUTs), pyruvate dehydrogenase kinase 1 (PDK1), erythropoietin, insulin-like growth factor 2 (IGF-2), transforming growth factor α, B-cell lymphoma 2 (BCL-2), BCL-2 interacting protein 3 (BNIP3), and heme oxygenase-1 (HO-1)^23^. In neuroblastoma cells, HIF-1α and HIF-2α reportedly share several target genes, such as VEGF, tyrosine hydroxylase, and N-myc downstream regulated 1^20^. In addition to these shared targets, specific target genes have been reported. For instance, BNIP3 expression solely depends on HIF-1α, while octamer-binding transcription factor 4 (OCT4) expression relies on HIF-2α^24,25^.

### O_2_ excess, oxidative stress, and NRF2

The transcription factor NRF2, encoded by the *NFE2L2* gene, plays a crucial role as a master regulator in maintaining cellular redox homeostasis in response to oxidative stress, which can be induced by excess O_2_^5,26^. Upon activation, NRF2 triggers the expression of hundreds of genes involved in various cellular processes, including antioxidant and xenobiotic responses, cell proliferation and survival, and metabolism, among others^9,27^. Although H_2_O_2_ production increases in the lungs of rats exposed to hyperoxia, increased antioxidant enzyme expression delays H_2_O_2_-induced lung damage^28^. Subsequently, NRF2 was revealed to be the primary transcription factor responsible for this enhanced response system^29^. Similarly, hyperoxic incubation of pulmonary epithelial cells activated the NRF2 pathway via the ROS-epidermal growth factor receptor-phosphoinositide-3-kinase (PI3K) signaling axis^30^.

NRF2 is a cap’n’collar (CNC) leucine zipper (bZIP) transcription factor consisting of seven Neh domains, each with distinct functions^5^. The Neh1 domain contains the CNC-bZIP region for DNA binding and heterodimerization with small MAF (sMAF) proteins. The Neh2 domain of NRF2 contains highly conserved DLG and ETGE motifs, which bind to its negative regulator Kelch-like ECH-associated protein 1 (KEAP1)^31^. The binding of the Neh2 domain of NRF2 to the Kelch domain of KEAP1 is the primary mechanism of NRF2 activity regulation^5,9^. The Neh3–5 domains play a role in transactivation activity through binding to multiple components of the transcriptional machinery. The Neh6 domain mediates KEAP1-independent negative regulation of NRF2 stability through binding to β-transducing repeat-containing protein (β-TrCP) (Fig. 1, right panel)^32^.

NRF2 activity is tightly regulated by the KEAP1-mediated turnover process. Under normal conditions, NRF2 is bound to two molecules of KEAP1, an adapter protein for CUL3-based E3 ubiquitin ligase, and is continuously degraded by the 26S proteasome^5^. Under oxidative stress conditions, ROS or electrophiles interact with or modify KEAP1 cysteine (Cys) residues, resulting in conformational changes in the KEAP1 protein and subsequent disruption of the NRF2 DLG motif-KEAP1–CUL3 complex. This process eventually leads to the nuclear translocation of newly synthesized NRF2 and the binding of the NRF2–sMAF complex to antioxidant response elements (AREs; 5′-A/GTGACnnnGC-3′), resulting in the transactivation of target genes (Fig. 1, right panel)^33^. The interaction between NRF2 and KEAP1 involves two models based on KEAP1 responses. The first model is the Cys sensor-dependent mechanism, where Cys residues in KEAP1 sense oxidizing or electrophilic signals^34^. For example, Cys151 in the BTB domain responds to sulforaphane, while Cys226/613/622/624 are identified as H_2_O_2_-responding sensors for NRF2 activation^33,35^. The second model is the Cys-sensor-independent mechanism, where KEAP1 function is hindered by protein–protein interactions rather than being dependent on changes in Cys residues. The ETGE motif in NRF2 strongly binds to KEAP1, acting as a hinge, while the DLG motif functions as a latch due to its decreased binding affinity. P62, which has a greater affinity for KEAP1 than the DLG motif in NRF2, acts as a protein–protein interaction inhibitor. Consequently, increased p62 levels can disrupt NRF2 DLG-KEAP1 binding, leading to the activation of NRF2 target gene expression^36^.

NRF2 is involved in the regulation of more than 200 genes. These genes encode several phase I- and II-metabolizing enzymes, including NAD(P)H: quinone oxidoreductase 1 (NQO1), aldo-keto reductases, glutathione (GSH) S-transferases, and UDP glucuronosyltransferases. They also encompass phase III drug efflux transporters and components of the GSH-based system, including glutamate-cysteine ligase catalytic (GCLC) and modifier subunits (GCLM), GSH reductase, and GSH peroxidases (GPXs). Additionally, NRF2 regulates genes related to thiol proteins, including thioredoxins (TRXs) and peroxiredoxins, as well as genes involved in carbohydrate metabolism and NADPH-generating enzymes such as glucose-6-phosphate dehydrogenase (G6PD) and malic enzyme 1. NRF2 also influences genes associated with heme and iron metabolism, such as HO-1 and ferritins, as well as genes encoding signaling molecules and transcription factors^37^. This regulatory mechanism is crucial for maintaining the cellular redox balance and defending against oxidative stress-related damage.

### Cross response: hypoxia-NRF2 and oxidative stress-HIFs

In real-world situations, the coexistence and interplay of hypoxia and oxidative stress result in a more complex response of HIFs and NRF2. It has long been established that hypoxic conditions lead to an increase in oxidative stress markers, including oxidized GSH. Consistent with this, increases in antioxidants, such as GPX and superoxide dismutase, have been reported in various animal models of hypoxia^10^. These observations suggest that ROS production increases under hypoxic conditions; therefore, determining both the stage of hypoxia and the mechanisms through which this increase occurs has been of interest. In most experimental hypoxia settings, ROS elevation has been a rapid response^10^; superoxide levels transiently increase within 10 min following acute hypoxia in human cells^38^. The involvement of mitochondria in ROS generation has been confirmed in several studies. Mitochondria-depleted Hep3B cells cannot produce ROS during hypoxia^39^. The transient superoxide burst induced by hypoxia also relies on functional mitochondria^38^. In particular, mitochondrial complex III has been identified as a mechanism for transient mitochondrial ROS generation under hypoxic conditions. Silencing of the Rieske iron-sulfur protein of complex III reduced hypoxia-induced ROS generation, suggesting the potential role of complex III as a hypoxic sensor^40^. Hypoxia-induced transient mitochondrial ROS are known to function as signaling molecules for cell proliferation and HIF activation^41^.

Under sustained hypoxic conditions, excess ROS accumulation can be harmful to cells. To protect mitochondria from oxidative stress, HIF-1α increases PDK1 expression levels, thereby activating mitochondria-independent metabolism. Moreover, HIF-1α enhances electron transfer efficiency by replacing the complex IV subunit and reducing ROS generation through the inhibition of complex I^12,42^. These actions help to prevent excessive mitochondrial ROS production during hypoxia. Despite the transient increase in ROS and antioxidants under hypoxic conditions, NRF2 activation has not been consistently observed. For instance, NRF2 levels in pulmonary epithelial and colorectal cancer cells and the rat retina remain unchanged or are reduced during hypoxia^43–45^, suggesting that other transcription factors activated by hypoxia, such as nuclear factor kappa B (NF-κB) and forkhead box O3 (FoxO), primarily lead to increased antioxidant protein expression levels^10^. Additionally, activation of HIF-1α reduces NRF2 transcriptional activity in ischemic mouse kidneys and vascular endothelial cells^46,47^.

Moreover, oxidative stress serves as an additional stimulator of HIF activation. Specifically, PHDs function as ROS sensors. PHD2, the primary HIF-1α hydroxylase, retains several Cys residues in its catalytic domain, and oxidative stress induces the dimerization of PHD2 through Cys oxidation, leading to the inhibition of PHD2 activity^11^. PHDs require Fe^2+^ as a cofactor, and the redox status of this cofactor is also crucial for their enzymatic activity. Depletion of the cellular antioxidant ascorbate leads to the oxidation of Fe^2+^ to Fe^3+^ in PHDs, resulting in the inactivation of these enzymes^48^. Additionally, the formation of an oxidant-induced GSH-Cys520 adduct in the HIF-1α protein promotes HIF-1α stabilization^49^. These lines of evidence collectively suggest HIF activation occurs in response to oxidative stress. Consistent with these findings, mitochondrial ROS, which increases during hypoxia, reportedly contributes to HIF upregulation^39,50^. These results showed that hypoxia and oxidative stress are linked factors rather than independent factors. Therefore, the effects of these two systems on HIFs and NRF2 signaling systems are complex, implying the presence of a network connecting these two systems.

### The interplay between HIFs and NRF2

**Fig. 2:**
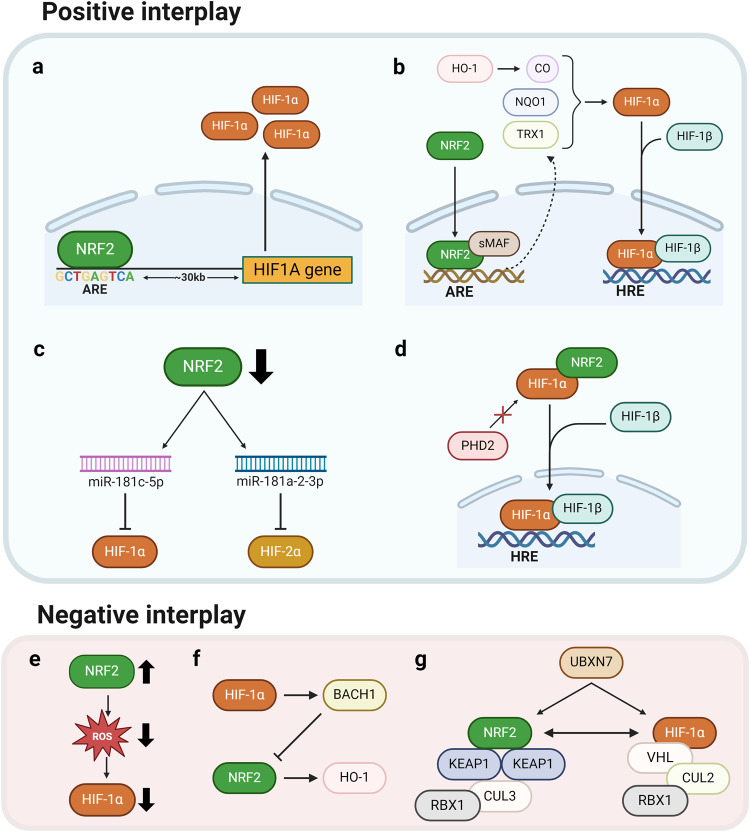
Interplay between HIFs and NRF2. [Top] Positive associations between HIFs and NRF2. **a** As a direct regulatory mechanism, NRF2 binds to the ARE located in the promoter region of the *HIF1A* gene, inducing the expression of HIF-1α. **b** The NRF2 targets TRX1 and NQO1 enhance the accumulation and stabilization of HIF-1α. Carbon monoxide produced from HO-1 also contributes to HIF-1α stabilization. **c** Inhibition of NRF2 increases the expression levels of miR-181c-5p and miR-181a-2-3p, suppressing HIF-1α and HIF-2α levels, respectively. **d** NRF2 can directly bind to the oxygen-dependent degradation domain of HIF-1α and prevent the interaction of HIF-1α with PHD2. [Bottom] Negative associations between HIFs and NRF2. **e** Increased NRF2 levels can suppress HIF-α through reduced ROS levels. **f** HIF-1α can repress the expression of NRF2 and HO-1 through the elevation of BACH1, a repressive partner of NRF2. **g** UBXN7 is a cofactor for the ubiquitination of both HIFs by CUL2- and NRF2 via CUL3-based complexes. UBXN7 has opposing effects on HIFs and NRF2. For instance, when UBXN7 is knocked out, NRF2 levels increase while simultaneously leading to a decrease in HIF-1α levels.

Considerable evidence suggests a correlation between the function and regulation of HIFs and NRF2. As indicated in Table 1, multiple reports suggest a positive association between HIFs and NRF2, which is often observed in cancer cells. In multiple cancer cell models, *NRF2* silencing has been shown to reduce HIF-1α levels, resulting in the suppression of HIF-1α-mediated cell proliferation, angiogenesis, tumor growth, and migration/invasion^45,51–55^. The positive relationship between HIF-1α and NRF2 has been explained through several mechanisms. First, as a direct regulatory mechanism, a conserved functional ARE was identified in the promoter region of the *HIF1A* gene (Fig. 2a). In this context, NRF2-mediated elevation of HIF-1α has been linked to increased levels of both NRF2 and HIF-1α in breast and bladder cancers, as well as cisplatin resistance in hepatocarcinoma cells (HCCs)^56,57^. Second, NRF2 target genes have been proposed to constitute a molecular link between HIF-1α and NRF2 (Fig. 2b). For example, NQO1 can directly bind to HIF-1α and subsequently prevent PHD binding and proteasomal degradation, leading to HIF-1α accumulation^58^. The NRF2 target TRX1 has been shown to increase HIF-1α activity^59^. In adenocarcinoma cells following intermittent hypoxia, NADPH oxidase 1 (NOX1)-induced ROS increases NRF2/TRX1 levels and thereby increase HIF-1α protein levels^60^. Carbon monoxide (CO), a major metabolite of HO-1, has been reported to stabilize the HIF-1α protein via translational suppression of HIF-1α ubiquitination^61,62^. Third, several signaling molecules have been suggested to act as intermediaries in the relationship between HIF-1α and NRF2. In glioblastoma, mitochondrial Nip3-like protein X (NIX), whose expression levels are increased by NRF2, promotes HIF elevation, resulting in the maintenance of glioblastoma stem cells^63^. In breast and colorectal cancer cells, *NRF2* silencing increased miR-181c-5p expression levels, subsequently suppressing mitochondrial respiration and O_2_ consumption by targeting the complex IV subunit, ultimately preventing HIF-1α accumulation under 24-h hypoxic conditions (Fig. 2c)^64,65^. Fourth, a direct interaction between the HIF-1α and NRF2 proteins has been reported (Fig. 2d). NRF2 directly binds to HIF-1α through the O_2_-dependent degradation domain; this interaction prevents PHD association, resulting in HIF-1α accumulation in pseudohypoxic HCC^55^. Most studies on the correlation between HIFs and NRF2 have focused primarily on HIF-1α, but an association with HIF-2α has been recently reported. HIF-2α levels, which gradually increase under continuous hypoxic conditions (72 h), decline following *NRF2* silencing and treatment with a chemical NRF2 inhibitor. In this context, NRF2 inhibition leads to the upregulation of miR-181a-2-3p, which directly targets HIF-2α, consequently resulting in the attenuation of the HIF-2α-mediated CSC phenotype in colorectal cancer cells (Fig. 2c)^66^.

**Table 1:** The positive association between HIFs and NRF2 in cancer underscores their collaborative roles in promoting tumor growth and progression.

Cancer	Mechanism	Phenotypes	Ref
Breast cancer	Single N-glycan deletion (N418Q) of ErbB3 protein (HER3) attenuates heregulin-β1-mediated nuclear accumulation of HIF-1α and NRF2 protein.	Cell growth, migration	^101^
	NRF2 knockdown decreases HIF-1α and subsequent genes involved in glycolysis.	Proliferation	^98^
	NRF2 binds to the ARE at 30 kilobases upstream of *HIF1A* locus.		^57^
	Overexpression of NRF2 promotes the expression of key enzymes in PPP, including G6PD and TKT, and subsequent HIF-1α and Notch1 signaling.	Proliferation, migration	^99^
	NRF2 knockdown increases miR-181c levels to inhibit HIF-1 α-mediated glycolysis and PPP metabolic adaptation and autophagy under hypoxia.	Metabolic reprogramming, autophagy	^65^
Colorectal cancer	NRF2 knockdown reduces cellular and mitochondrial O_2_ consumption and ATP production, thus impairing HIF-1α stabilization under hypoxia.	Tumor growth, angiogenesis	^45^
	NRF2, HIF, and NF-κB are enriched in the core of growing spheroids.	CSC, stress resistance	^165^
	NRF2 knockdown increases miR-181a-2-3p levels and inhibits HIF-2α stabilization under chronic hypoxia (1% O_2_, 72 h).	Tumor growth, CSC properties	^66^
Endometrial cancer	miR-148b decreases NRF2 and HIF-1α by downregulating ERMP1 under hypoxic conditions.	Proliferation	^192^
Esophageal squamous carcinoma	NRF2 shRNA blockade HIF-1α activation after CoCl_2_ treatment.	Migration, invasion	^53^
Gastric cancer	Hypoxia or VEGFA increases the expression of VEGFR2 to facilitate NRF2 nuclear translocation, while knockdown of NRF2 inhibited HIF-1α levels.	Survival, invasion	^100^
	TRPM2 silencing destabilizes NRF2 and HIF-1α through their ubiquitination.	Ferroptosis	^184^
Glioblastoma	NRF2 inhibition suppresses mitochondrial O_2_ consumption and thereby promotes HIF-1α degradation under hypoxic conditions.	Proliferation, tumor growth, angiogenesis	^52^
	Oxidative stress-induced NRF2 transactivation activates NIX and increases the level of HIF-1α and HIF-2α under hypoxic conditions.	CSC, survival, tumor growth	^63^
	Hypoxia (12 h) induces HIF-1α stabilization and promotes M2-polarized macrophages to activate the PI3K/Akt/NRF2 pathway.	Proliferation, CSC, EMT, drug resistance, tumor growth, angiogenesis	^164^
Hepatocellular carcinoma	NRF2 regulates the transcript level of *HIF1A via* ARE consensus on its promoter under mild hypoxic (5% O_2_) condition.	Cisplatin resistance	^56^
	NRF2 inhibition reduces HIF-1α/HSP70 signaling activation.	5-FU resistance, cell viability, migration, invasion, tumor growth	^112^
	NRF2 binds to the oxygen-dependent degradation (ODD) domain of HIF-1α and impairs the hydroxylation of HIF-1α by PHD2.	Tumor growth	^55^
Renal carcinoma	FH-deficient cells accumulate fumarate to stabilize ROS-independent NRF2 and ROS-dependent HIF-1α.	Proliferation	^67^
Lung adenocarcinoma	Intermittent hypoxia enhances NOX1-mediated ROS production to activate NRF2 by protein stabilization and subsequent Trx1, which increases HIF-1α signaling.	Survival	^60^
Lung carcinogenesis	iAs induces NRF2-dependent HIF-1α activation to promote hexosamine biosynthesis and the serine/glycine pathway of glycolysis.	CSC, metabolic reprogramming	^51^
Ovarian cancer	Increases in ROS by follicle-stimulating hormone (FSH) activate NRF2, which further enhances HIF-1α-induced VEGF expression.	Angiogenesis	^54^
Pancreatic and lung cancer	Depletion of NRF2 impairs HIF-1α under hypoxia. Co-IP experiments revealed a protein interaction between NRF2 and HIF-1α.	Proliferation	^193^

*ARE* antioxidant response element, *Co-IP* coimmunoprecipitation assay, *CSC* cancer stem cell, *EMT* epithelial-to-mesenchymal transition, *ERMP1* endoplasmic reticulum metallopeptidase 1, *FH* fumarate hydratase, *G6PD* glucose-6-phosphate dehydrogenase, *HSP70* heat-shock protein 70, *NOX1* NADPH oxidase 1, *PPP* pentose phosphate pathway, *TKT* transketolase, *TRPM2* transient receptor potential cation channel, subfamily M, member 2, *TRX1* thioredoxin 1, *VEGF* vascular endothelial growth factor.

HIFs and NRF2 do not always positively correlate. For instance, under certain experimental conditions or in specific cell types, their relationship exhibits an inverse association. For example, there are reports describing the regulation of HIFs and NRF2 in terms of changes in ROS (Fig. 2e). For instance, in fumarate hydratase-deficient cancer cells, accumulated fumarate results in the production of succinate GSH, resulting in increased mitochondrial ROS and subsequent HIF-1α activation. In this context, fumarate-induced NRF2 contributes to HIF-1α inhibition; therefore, *NRF2* knockdown in these cells further increases HIF-1α levels^67^. The molecular clock protein BMAL1 directly upregulates NRF2 expression via E-box binding. Therefore, *BMAL1*-deficient cells exhibit increased HIF-1α expression levels due to ROS accumulation, which results from a reduction in NRF2 expression levels^68^. More specific causal relationships have been reported. HIF-1α induction repressed the NRF2 transcription of HO-1 and interleukin-8 in endothelial cells; the decreased NRF2 activity was found to be due to the elevation of BTB and CNC homology 1 (BACH1), a repressive partner of NRF2 (Fig. 2f)^47^. The reciprocal regulation of HIFs and NRF2 is reportedly attributed to a ubiquitination process involving these proteins (Fig. 2g). UBXN7 is a cofactor that provides a scaffold for both the ubiquitination of HIFs by a CUL2-based E3 ligase and that of NRF2 by a CUL3-based E3 ligase. Elevated UBXN7 levels are associated with HIF-1α accumulation, while *UBXN7* knockout results in increased NRF2 levels and HIF-1α downregulation^69^. In line with these reports of a negative association, *NRF2* silencing reinforced HIF-1α accumulation in alcohol-treated liver tissue^70^ and ischemia‒reperfusion-treated lung tissue^71^. In this context, treatment with several types of NRF2-activating agents suppresses HIF levels. Treating human endothelial cells with andrographolide suppressed HIF-1α, and NRF2 activation was involved in this process^72^.

## O_2_ response systems and cancer

### Elevated HIFs and NRF2 in cancer

In advanced solid tumors, hypoxic regions arise due to abnormalities in the tumor vasculature; hence, intratumoral hypoxia is the principal stimulus for HIF activation^2^. In support of this, immunohistological analysis of human cancer biopsies has shown that cancer cells exhibit higher HIF expression levels than their surrounding normal cells^23,73^. Tumor cells express HIF-regulated target genes that are critical for cancer progression, including those involved in tumor angiogenesis, metabolic shifts, metastasis/invasion, therapeutic resistance, and immune invasion^74^. Recent findings have also revealed the significant role of HIF-2α in cancer stemness^19^.

In some tumor tissues, HIFs are evenly expressed regardless of blood vessel formation, suggesting the occurrence of an O_2_-independent mechanism for HIF elevation. One well-characterized example is a loss-of-function mutation of the *pVHL* gene and subsequent elevation of HIF-1α and HIF-2α in clear cell renal cell carcinomas^75^. Additional associations between tumor suppressor gene deletion and HIF elevation have been identified. Loss of the p53 tumor suppressor impairs MDM2-mediated HIF-1α degradation by the proteasome, thus enhancing the transcriptional activation of VEGF^76^. Dominant-negative mutations in the tumor suppressor phosphatase and tensin homolog (*PTEN*) increase HIF-1α expression levels, leading to target gene expression in prostate cancer^77^. Fumarate, an oncometabolite that accumulates in fumarate hydratase-deficient renal cell carcinoma, induces HIF upregulation by competing at the PHD^78^. In addition, hypoxia-induced miRNAs, termed hypoxamiRs, regulate HIFs and subsequent cell responses to hypoxia^79^.

NRF2 prevents cancer initiation by orchestrating protective mechanisms against potential carcinogens and other hazards in normal and healthy cells. However, in cancer cells, NRF2 has oncogenic effects, contributing to tumor growth and progression^9,80^. Within the TME characterized by oxidative conditions, the delicate balance of NRF2 regulation is frequently disrupted, resulting in aberrant NRF2 activation^9,31,80^. Dysfunction of the NRF2 pathway leads to the activation of cellular defense systems and subsequent target gene upregulation, thereby promoting the survival and proliferation of cancer cells.

*NRF2* mutations are classified as gain-of-function mutations and are concentrated in the DLG and ETGE motifs responsible for KEAP1 binding. These mutations disrupt the interaction between NRF2 and KEAP1. This prevents the formation of the NRF2-KEAP1-CUL3 complex required for NRF2 degradation, resulting in NRF2 accumulation and constitutive activation of its target genes^80,81^. Mutations in *KEAP1* are classified as loss-of-function mutations and are distributed throughout the entire *KEAP1* genome, with particular enrichment observed within the Kelch domain, which interacts with the DLG and ETGE motifs of NRF2^9,35,80^. In these mutant cancer cells, NRF2 is constitutively activated, upregulating target genes that promote cancer cell survival, proliferation, progression, and therapeutic resistance^81,82^. Mutations in *NRF2* and *KEAP1* have been identified in multiple cancers with varying prevalence. Somatic mutations in *NRF2* were found in 6% of patients with HCC^83^, and *KEAP1* mutations have been reported to be present in 30% and 20% of patients with gallbladder and lung adenocarcinomas, respectively^84,85^. In addition to somatic mutations in *NRF2* and *KEAP1*, several molecular mechanisms have been elucidated for NRF2 overactivation in cancers, including *KEAP1* silencing through promoter methylation^86^, transcriptional NRF2 activation by oncogenes such as KRAS^87^, NRF2 stabilization by p62 accumulation^88^, and NRF2 activation by the oncometabolite fumarate^89^.

**Fig. 3:**
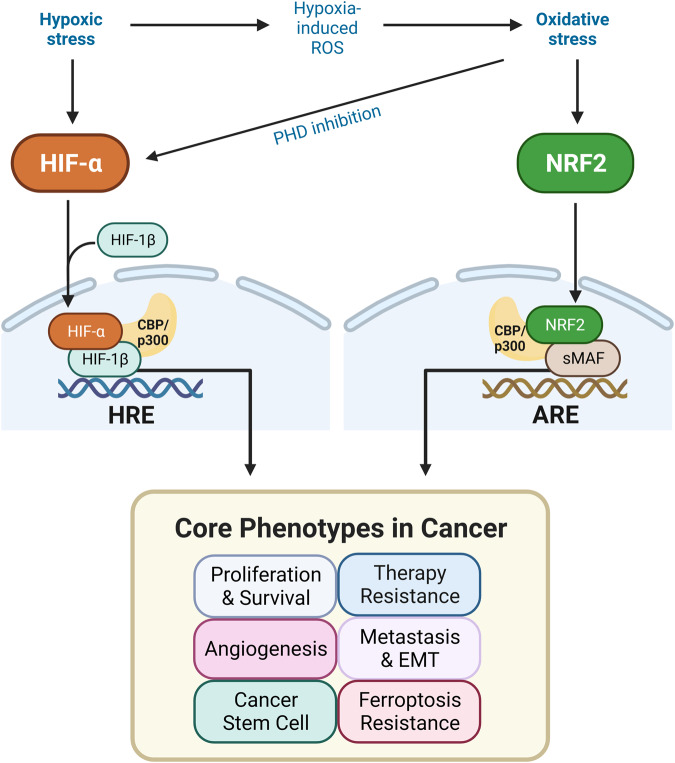
Cooperative effects of HIFs and NRF2 on core cancer phenotypes. In the tumor microenvironment characterized by hypoxia and oxidative stress, HIFs, and NRF2 are aberrantly activated, promoting core cancer phenotypes, such as cancer proliferation and survival, therapeutic resistance, angiogenesis, EMT/metastasis, CSC trait acquisition, and resistance to ferroptosis. HIFs respond to both hypoxia and oxidative stress, which are likely inevitable conditions under low-oxygen conditions. In contrast, changes in NRF2 under hypoxic conditions vary in a context-dependent manner, indicating that hypoxia-associated oxidative stress does not necessarily accompany NRF2 activation.

Activated HIFs and NRF2 in cancer cells can cooperatively promote tumor growth and progression by upregulating common target genes (Table 2). Consistent with these findings, HIF-1α and NRF2 expression levels are mutually associated, and high levels of these transcription factors correlate with poor outcomes in glioblastoma patients^90^. In the following sections, we investigate the specific roles of HIFs and NRF2 in the fundamental characteristics of cancer, including cancer growth and survival, therapeutic resistance, angiogenesis, metastasis, CSCs, and ferroptosis resistance (Fig. 3). Specifically, our emphasis will be on scrutinizing the interactions between HIFs and NRF2 concerning these fundamental aspects of cancer.

**Table 2:** The target genes of HIFs and NRF2 are involved in cancer proliferation/survival, therapy resistance, angiogenesis, EMT/metastasis, cancer stem cell trait acquisition, and ferroptosis.

Cancer phenotypes	NRF2 target genes	HIFs target genes
Proliferation/Survival/Metabolic shift	***NOTCH1***^194^, ***HK2***, ***PKM***^195^, *G6PD, PGD, ME1*^37^	***NOTCH1***^196^, ***HK2***, ***PKM*** *PFKFB3, LDHA*, *PDK1 ALDOA*, *PGK1*, *PFKL*^2^
Therapy resistance	***BCL-2***, ***ABCC1***, ***ABCG2***, *ABCC2, ABCC3*^107^	***BCL-2***^104^, ***ABCC1***^197^, ***ABCG2****, ABCB1*^2^
Angiogenesis	***VEGF***^45^, *HMOX1*^37^	***VEGF***, *SDF1, SCF, PGF, ANGPT2*^2^
EMT/Metastases	***NOTCH1***^194^, ***HMOX1***^37^, *NOTCH4*^133^	***NOTCH1***^196^, ***HMOX1***^198^, *MMP2, ZEB1*, *SNAI1*, *TWIST*, *SNAI2 (SLUG)*, *ADAM12*^2^
Cancer stem cells	***OCT4, NANOG***^199^, ***NOTCH1***^194^, ***SLC7A11***^37^, ***ABCB1, ABCG2****, ABCC1*^107^	***OCT4***^24^, ***NANOG****, SOX2*^200^, ***NOTCH1***^196^, ***SLC7A11, ABCB1, ABCG2****,* CD47, *CXCR4*^2^
Ferroptosis	***SLC7A11***, ***GCLM***, ***HMOX1****, GCLC, FPN, FTL, FTH1, TXNRD1, GPX4, NQO1*^177^, *HERC2, VAMP8*^182^	***SLC7A11, GCLM***^2^, ***HMOX1***^71^

*NOTCH1* neurogenic locus notch homolog protein 1, *HK2* hexokinase-2, *PKM* pyruvate kinase muscle isozyme, *G6PD* glucose-6-phosphate dehydrogenase, *PGD* phosphogluconate dehydrogenase, *ME1* malic enzyme-1, *PFKFB3* 6-phosphofructo-2-kinase/fructose-2,6-biphosphatase 3; *LDHA* lactate dehydrogenase A, *PDK1* pyruvate dehydrogenase kinase 1, *ALDOA* aldolase A, *PGK1* phosphoglycerate kinase 1, *PFKL* 6-phosphofructokinase, liver type, *BCL-2* B-cell lymphoma 2, *ABCC1* ATP binding cassette subfamily C member 1, *ABCG2* ATP-binding cassette superfamily G member 2, *ABCC2* ATP binding cassette subfamily C member 2, *ABCC3* ATP binding cassette subfamily C member 3, *ABCB1* ATP binding cassette subfamily B member 1, *HMOX1* heme oxygenase 1, *MMP2* matrix metallopeptidase 2, *ZEB1* zinc finger E-box-binding homeobox 1, *SNAI1* snail family transcriptional repressor 1, *TWIST* twist family BHLH transcription factor 1, *ADAM12* a disintegrin and metalloprotease 12, *OCT4* octamer-binding transcription factor 4, *NANOG* NK2-family homeobox transcription factor, *SLC7A11* solute carrier family 7 member 11 (xCT), *SOX2* SRY (sex determining region Y)-box 2, *CD47* cluster of differentiation 47, *CXCR4* C-X-C chemokine receptor type 4, *GCLM* glutamate–cysteine ligase modifier subunit, *GCLC* glutamate–cysteine ligase catalytic subunit, *FPN* ferroportin-1, *FTL* ferritin light chain, *FTH1* ferritin heavy chain, *TXNRD1* thioredoxin reductase 1, *GPX4* glutathione peroxidase 4, *NQO1* NAD(P)H quinone dehydrogenase 1, *HERC2* HECT domain and RCC1-like domain 2, *VAMP8* vesicle associated membrane protein 8.

### Cancer proliferation and survival

HIF-1α plays a critical role in cancer survival and proliferation in hypoxic environments by regulating a wide range of genes. First, HIF-1α promotes glycolysis by upregulating key glycolytic enzymes, including aldolase A, phosphoglycerate kinase 1, and pyruvate kinase M. Similarly, HIF-1α inhibits mitochondrial oxidative phosphorylation through the upregulation of lactate dehydrogenase A and PDK1^91,92^. Additionally, HIF-1α elevates GLUT4 levels to increase cellular glucose uptake^93^. This metabolic shift enables cancer cells to produce ATP with reduced ROS generation under O_2_-deprived conditions, ultimately promoting cancer proliferation. Second, as an adaptive stress resistance mechanism, HIF-1α activates autophagy by upregulating BNIP3, facilitating the removal of damaged organelles such as mitochondria^94^. Third, HIF-1α suppresses protein synthesis by modulating multiple genes involved in the translation process, thereby reducing energy expenditure and preventing unfolded protein accumulation^95^.

In parallel, NRF2 participates in cancer proliferation and survival through downstream target genes. First, antioxidant and detoxification systems, such as GSH synthesis and regenerating enzymes, protect cancer cells against the oxidizing tumor environment and cytotoxic anticancer drug treatment^96^. Second, the elevation of pentose phosphate pathway genes, including G6PD and 6-phosphogluconate dehydrogenase, facilitates purine biosynthesis and NAD(P)H generation, resulting in enhanced cell proliferation^37^. Third, NRF2 has been shown to regulate the expression of genes associated with cell growth, including IGF-1 and bone morphogenetic protein receptor 1 (BMPR1), as revealed by ChIP-seq profiling analysis^97^.

Multiple lines of evidence support the collaborative interplay between NRF2 and HIF in promoting cancer proliferation and survival. Research conducted on breast cancer cells demonstrated the cooperative effect of upregulated NRF2 and HIF-1α on cell proliferation, which led to the enhancement of glycolytic genes, including hexokinase 2, pyruvate kinase M2, and lactate dehydrogenase A^98^. Targeting *NRF2* with shRNA resulted in the downregulation of HIF-1α and glycolytic genes, ultimately leading to the suppression of cancer proliferation. The upregulation of NRF2 stimulates the expression of G6PD and activates HIF-1α, resulting in NOTCH1 signaling activation and breast cancer proliferation^99^. Similarly, in HCC, elevated NRF2 and HIF-1α levels contribute to cancer growth and progression. *NRF2* knockdown mitigated HIF-1α accumulation, while *HIF-1α* silencing did not affect NRF2 status. Mechanistically, a direct interaction between NRF2 and HIF-1α could impede PHD-VHL-mediated degradation, thus contributing to HIF-1α stabilization^55^. In tissue specimens from patients with gastric cancer, high NRF2 expression levels were positively correlated with HIF-1α and HO-1 expression^100^. In vitro, hypoxic conditions increased NRF2 and HO-1 expression levels, as did HIF-1α. *NRF2* knockdown blocked HIF-1α and HO-1 upregulation, further suppressing gastric cancer survival. Mechanistically, hypoxia-driven VEGF-A activates the nuclear translocation of NRF2 through the RAP1B/ERK/AKT signaling cascade^100^. The significance of AKT signaling in the regulation of HIFs and NRF2 has also been demonstrated in ERBB3-induced breast cancer growth^101^. The *ERBB3* mutant protein (ERBB3 N418Q) blocked HIF-1α and NRF2 accumulation by inhibiting PI3K-AKT signaling activation, which resulted in the suppression of cancer growth and migration. These results demonstrate that in many tumors, HIFs and NRF2 jointly contribute to cancer cell proliferation and survival, with increases in NRF2 often directly or indirectly linked to increases in HIFs.

### Therapeutic resistance

Increased anticancer drug efflux is one of the major mechanisms by which cancer cells mediate multidrug resistance and therapy refractoriness. HIF-1α promotes the expression of ATP-binding cassette (ABC) transporters, including multidrug resistance 1 (MDR1), multidrug resistance-associated protein 1 (MRP1), and breast cancer resistance protein (BCRP), by acting on HREs^102,103^. Additionally, HIF-1α induces the expression of antiapoptotic proteins, such as BCL-2 and BCL-XL, while inhibiting the expression of proapoptotic proteins, such as BCL-2-associated X protein (BAX) and BH3 interacting domain death agonist (BID)^104–106^. These changes consequently result in coordinated regulation that promotes cancer cell survival by preventing apoptotic cell death, further contributing to drug resistance.

The constitutive activation of NRF2 in cancer is a significant factor that enhances the elimination of anticancer drugs by increasing the expression levels of phase II metabolizing enzymes, leading to chemotherapy resistance^107^. In addition, NRF2 promotes drug efflux by upregulating multiple ABC transporters, such as MDR1 and BCRP^107,108^. Functional AREs have been characterized in the promoter regions of these ABC transporters^109,110^. Furthermore, NRF2-mediated elevations in the levels of the antiapoptotic protein BCL-2 are involved in resistance to etoposide-induced cell death^111^.

Several studies have shed light on the link between NRF2 and HIFs in conferring resistance to anticancer therapy. Under mildly hypoxic conditions (5% O_2_) in HCC, NRF2 can directly interact with the ARE located at the 5′ upstream region of the *HIF1A* gene^56^. This interaction leads to the upregulation of HIF-1α expression, thereby resulting in resistance to cisplatin treatment. In 5-fluorouracil-resistant HCC, *NRF2* knockdown decreases HIF-1α expression levels, while its overexpression increases HIF-1α expression levels^112^. In this context, the administration of siRNA targeting *NRF2* enhances the anticancer effect of As_2_O_3_ by inhibiting HIF-1α/HSP70 signaling and subsequently inhibiting BCL-2. These studies suggest that NRF2 and HIF-1α cooperate in the development of chemotherapy resistance.

### Tumor angiogenesis

Angiogenesis occurs as tumors grow. It involves the release of proangiogenic factors that stimulate tumors to progress from dormancy and lead to the formation of new blood vessels, inducing rapid growth^113^. One well-established proangiogenic factor is VEGF. Under hypoxic conditions, tumor cells invade neighboring tissues from primary sites and metastasize through tumor neovascularization, which is regulated by VEGF. A HIF-1 binding site has been identified and characterized in the *VEGF* gene, and *ARNT*-deleted cells failed to increase VEGF expression levels upon exposure to hypoxic stimuli^114^. Additionally, other angiogenic growth factors, including stromal-derived factor 1, stem cell factor, and angiopoietin family members, are also regulated by HIFs and participate in tumor vascularization^115–117^.

NRF2 has been reported to increase the expression of several angiogenic target genes, including HO-1, VEGF, and IGF-1; therefore, its activity can influence the blood vessel formation process^9,41^. The association between NRF2 and angiogenesis regulation has been demonstrated in studies using *Nrf2*^−/−^ mice^118,119^. In these studies, revascularization was promoted in *Nrf2*^−/−^ mice compared with wild-type mice in a model of hindlimb ischemia. However, in vitro, angiogenic cytokine treatment activates the nuclear translocation of NRF2, which stimulates the tube formation of endothelial cells. In addition, the survival and angiogenic response of bone marrow-derived proangiogenic cells from *Nrf2*^−/−^ mice were significantly reduced^114^. These discrepancies between in vitro cell systems and in vivo animal models can be attributed to the potential proangiogenic role of ROS and the increased inflammatory response in *Nrf2-*deleted mice^118^. Additional studies also suggest a positive role for NRF2 in angiogenesis. The specific deletion of *NRF2* in endothelial cells results in decreased vascular density and angiogenic sprouting by increasing delta-like ligand 4 expression and NOTCH activity^120^. Loss of *Nrf2* inhibited VEGF elevation in the brains of venous hypertensive rats, and *Nrf2* knockout suppressed vascular tube formation in primary brain microvascular endothelial cells^121^. A coculture model of HCC with monocytes revealed that NRF2 activation in macrophages induces an M2-like phenotype, promoting VEGF elevation in cancer cells and subsequent EMT^122^.

In this context, evidence suggests that *NRF2* silencing blocks HIF-1α signaling to suppress blood vessel formation and xenograft tumor growth^45,52,54^. In colon cancer cells, *NRF2* silencing impaired HIF-1α elevation, leading to angiogenesis suppression both in vitro and in vivo^45,64^. In addition to these molecular events, elevated miR-181c expression levels and a subsequent reduction in O_2_ consumption were suggested to promote HIF-1α protein degradation in NRF2-inhibited cells. Similarly, *NRF2* knockdown in glioblastoma and ovarian cancer cells led to reduced HIF-1α levels, with a concurrent reduction in VEGF expression levels^52,54^. However, curcumin supplementation upregulated NRF2 and GSH, thus inhibiting HIF-1α accumulation and ultimately suppressing HCC angiogenesis and invasion^123^.

### EMT and cancer metastasis

Hypoxia is a strong driving force for EMT, a process in which cancer cells undergo phenotypic conversion to a mesenchymal phenotype, enhancing their migration and invasion. The signaling pathways and transcription factors associated with EMT, including NF-κB, transforming growth factor β (TGF-β), SNAIL, and TWIST, are enhanced by hypoxia^124^. A preliminary study analyzing HIF-1α levels in tumor specimens showed that HIF-1α levels were elevated in 69% of metastatic breast cancers, while only 29% of primary breast cancers exhibited elevations in HIF-1α levels^125^. Consistent with these findings, the proximal promoter regions of *SNAIL*, *TWIST*, and *ZEB1* contain HREs that directly interact with HIF-1α under hypoxic conditions^126–128^. In breast cancer cells, elevated expression levels of a disintegrin and metalloproteinase 12, mediated by HIF-1α and HIF-2α, induce hypoxia-driven migration and invasion, resulting in breast cancer metastasis to the lung^129^.

Despite the clear involvement of HIFs in EMT and cancer migration, the impact of NRF2 on these aspects is not consistent. Cancer cells with constitutively high NRF2 levels exhibit activated EMT and cancer migration^130,131^. Overactivation of NRF2 by deleting the *KEAP1* gene in lung squamous cell carcinoma facilitates tumor metastasis^132^. NRF2 has also been linked to TGF-β1-induced EMT by directly upregulating NOTCH4 expression^133^. In this context, *NRF2* knockdown or ROS inhibition attenuated NOTCH signaling and subsequently suppressed EMT in lung cancer cells. Moreover, NRF2 was identified as a phenotypic stability factor for hybrid epithelial/mesenchymal and activated NOTCH signaling for cancer migration^134^. However, a negative role of NRF2 in EMT and cancer metastasis has been suggested. In HCC, loss of NRF2 increases cancer plasticity and motility by enhancing SMAD signaling^135^. *NRF2*-silenced lung cancer cells exhibit an increase in both basal and TGF-β-induced cell motility through increased ROS levels^136^.

Several studies have suggested a cooperative link between HIFs and NRF2 in cancer metastasis. NRF2 overexpression and *KEAP1* knockdown promoted the expression of G6PD and HIF-1α/NOTCH1 to induce EMT and breast cancer migration^99^. Hypoxic conditions increase NRF2, HIF-1α, and HO-1 expression in gastric cancer cells, whereas NRF2 inhibition suppresses cancer invasion via concomitant reductions in HIF-1α expression^100^. In a hypoxia-mimicking model using CoCl_2_, *NRF2* knockdown reduced HIF-1α, HO-1, and matrix metalloproteinase 2 expression levels, resulting in the suppression of cancer migration and invasion in esophageal squamous cell carcinoma^53^.

### Cancer stem cell traits

CSCs are a small subset of cells within a tumor that possess self-renewal and tumor-initiating capacities. The presence of CSCs is thought to cause cancer treatment failure, as they drive malignant properties, including metastasis, invasion, therapeutic resistance, and immune escape^137^. Several embryonic pluripotency markers, including SRY-box 2 (SOX2), Krüppel-like factor 4 (KLF4), OCT4, and NANOG, have been identified as CSC markers^138^. Specific CSC markers have been identified for each tumor type and are commonly used for the isolation and characterization of CSCs. For example, CD44^+^, CD24^−^, and CD133^+^ have been used to characterize breast CSCs^139^, while CD44^+^, epithelial cell adhesion molecule^high^, and CD166^+^ are used as colorectal CSC markers^140^.

Restricted O_2_ availability induces enrichment and dependency of CSCs on HIFs for survival, self-renewal, and growth^141^. In triple-negative breast cancer, chemotherapy increases the expression levels of HIF-1α and its target genes, subsequently leading to an increased number of breast CSCs through elevations in the levels of interleukins 6 and 8^142^. This study showed that HIF inhibitors can restore the sensitivity of breast CSCs to chemotherapy. Antiangiogenic agents, such as bevacizumab, induce CSC enrichment in breast cancer xenografts by creating an intratumoral hypoxic environment and upregulating HIF-1α expression^143^. As factors contributing to underlying molecular mechanisms, HIFs activate transcription factors and signaling pathways involved in CSC self-renewal and pluripotency, including OCT4 and NOTCH^144^. HIF-2α directly binds to the *OCT4* promoter and increases OCT4 expression levels, promoting teratoma growth^145^. Chemotherapy-induced HIF-1α upregulates the expression of S100A10, which forms a transcription complex at the OCT4 binding site to facilitate the expression of other pluripotency genes, including NANOG, SOX2, and KLF4^146^. In ovarian cancer, the HIF-1α-driven NF-κB pathway is responsible for inducing CSC properties through SIRT1 upregulation^147^. Moreover, evidence suggests that HIF-2α plays a role in maintaining CSC traits through the activation of WNT and NOTCH signaling^148^ by elevating superoxide dismutase (SOD) levels and by reducing mitochondrial reactive oxygen species (ROS) levels^149^. Consistent with these findings, *HIF-2α* knockdown in renal cell carcinoma inhibited the expression of CXCR4, a chemokine receptor used as a renal cell carcinoma stem cell marker, resulting in the inhibition of spheroid and tumor growth^150^. These studies collectively underscore the critical role of the HIF pathway in regulating CSC traits.

CSCs reportedly exhibit higher antioxidant gene expression levels and lower ROS levels than normal cells^151^. This finding suggests that NRF2 is involved in the development of CSC traits. Currently, accumulating evidence suggests that NRF2 signaling is involved in maintaining the stemness phenotype of CSCs^152^. Head and neck cancer cells displaying low ROS levels exhibit more CSC traits, such as stemness and chemoresistance. Elevated NRF2 levels are responsible for maintaining low-ROS CSCs by increasing the expression of antioxidant and glycolytic enzymes and stemness markers^153,154^. In sorafenib-resistant HCC, NRF2 signaling promoted CSC traits and increased ABC transporter expression levels^155^. In glioblastoma stem cells (GSCs) isolated from surgical specimens, *NRF2* silencing decreased the proportion of GSCs and the expression levels of self-renewal markers, including SOX2^156^. Chemotherapy-induced breast CSCs exhibit CD44 enrichment and CSC traits, including spheroid growth and increased tumorigenesis. These changes are mediated by p62-associated NRF2 activation^157^. Similarly, additional experiments involving the isolation of CSC fractions using aldehyde dehydrogenase, epithelial cell adhesion molecule, or CD133 revealed that increased NRF2 levels play a critical role in developing CSC traits and maintaining and promoting CSC growth^158–160^. In addition to its role in antioxidant and drug efflux systems, NRF2 supports CSC stemness traits through the upregulation of NOTCH, FOXO3, and β-catenin expression^161–163^. Although the exact underlying mechanism by which NRF2 expression increases in CSCs has not been fully elucidated, it has been suggested that increases in the levels of p62 or the endoplasmic reticulum stress-activated protein kinase R-like ER kinase (PERK) may be involved^153,157,159,160^.

Several reports have demonstrated an association between HIFs and NRF2 in CSCs. Lung epithelial cells exposed to inorganic arsenic develop CSC-like properties with increased expression levels of stemness markers. During this process, NRF2 activation stimulates increases in HIF-1α levels, allowing metabolic reprogramming toward glycolysis^51^. In this context, *NRF2* knockout diminished inorganic arsenic-induced HIF-1α accumulation and stemness marker expression. In clinical samples from patients with glioblastoma, elevated mitochondrial NIX expression levels were identified as a marker of GSCs, and hypoxia-induced elevations in NIX levels were linked to increased ROS-driven NRF2 expression, further contributing to CSC stemness through HIF/mTOR activation^63^. Hypoxia-induced M2 phenotype macrophages promote the stemness traits of glioblastoma through VEGF secretion, which is mediated by HIF-1α activation. PI3K/AKT-mediated NRF2 activation is involved in this VEGF-GSC axis^164^. In a study involving spheroid culture of colon cancer cells transfected with a reporter monitoring the transcriptional activity of HIFs and NRF2, increases in the expression levels of these transcription factors were observed within the spheroid cores, suggesting coordinated HIF and NRF2 regulation in a 3D culture system^165^. Additionally, in colorectal cancer cells, prolonged hypoxia-induced elevations in the levels of OCT4 and CSC traits are suppressed by NRF2 inhibition, and the underlying mechanism of this effect is the repression of HIF-2α accumulation^66^.

### Resistance to ferroptosis

Ferroptosis is an iron-dependent lipid peroxidation-mediated cell death pathway that involves characteristics distinct from those of apoptosis, such as an intact cell membrane and nucleus, increased bilayer membrane density, and reduced mitochondrial volume and cristae^166^. Increased intracellular free iron causes ROS generation through the Fenton reaction, resulting in lipid peroxide accumulation and subsequent ferroptosis^167^. In this context, the iron chelating agent RSL3 inhibits ferroptosis^168^. The removal of H_2_O_2_ by GPX4 is essential for preventing ferroptosis. *Gpx4* knockout in mice leads to ferroptosis-induced cell death in the kidney^169^. Emerging studies on ferroptosis in various diseases have revealed the association of this novel cell death system with pathological processes, including cancer. Treatment-resistant mesenchymal-type cancer cells are strongly dependent on GPX4, and lipoxygenase inhibition can block ferroptotic cell death via GPX4 inhibition^170^.

Hypoxia and HIFs are associated with ferroptosis in a context-dependent manner. Hypoxia decreases cellular free iron levels and increases ferritin expression levels, resulting in the protection of hypoxic macrophages against RSL3-induced ferroptosis^171^. In clear cell renal cell carcinoma, elevated HIF levels lead to the suppression of ferroptosis in response to erastin or GSH synthesis inhibition^172^. The pharmacological or siRNA-mediated inhibition of iron-sulfur cluster assembly 2 in mitochondria decreases HIF-1α and HIF-2α levels, resulting in subsequent iron overaccumulation and ferroptosis-mediated cell death in clear cell renal cell carcinoma^173^. Aryl hydrocarbon receptor nuclear translocator-like protein inhibits ferroptosis by activating HIF-1α in non-small cell lung cancer cells^174^. In contrast, some reports suggest that HIFs induce ferroptosis under certain conditions. HIF-2α activation in colorectal cancer upregulates genes involved in lipid and iron metabolism, resulting in enhanced susceptibility to dimethyl fumarate-induced ferroptosis^175^. Treatment of glioblastoma cells with the PHD inhibitor roxadustat results in the accumulation of lipid peroxides and iron, leading to ferroptosis^176^; in this case, HIF-2α contributes to ferroptosis by upregulating lipid regulatory genes.

NRF2 directly upregulates genes associated with iron metabolism (HO-1, ferritin light and heavy chain) and antioxidant activity (GPX4, thioredoxin reductase 1, GCLC, GCLM, and the cystine/glutamate antiporter SLC7A11), which prevent free iron accumulation and lipid peroxidation. Therefore, NRF2 has been recognized as a negative regulator of ferroptosis^177^. In glioblastoma cells, forced NRF2 expression upregulates SLC7A11 (also known as xCT) and promotes ferroptosis resistance^178^. NRF2 inhibition augments erastin-induced ROS generation via SLC7A11 reduction, thereby sensitizing glioma cells to ferroptosis. Another study involving head and neck cancer cells demonstrated that RSL3 resistance is associated with increased expression levels of NRF2 and p62; therefore, NRF2 or p62 inhibition reverses ferroptosis resistance^179^. Similarly, cetuximab treatment facilitates RSL3-induced ferroptosis through the inhibition of NRF2/HO-1 signaling in *KRAS*-mutant colorectal cancer cells^180^. Recently, ferroptosis suppressor protein 1 (FSP1) was identified as a novel target of NRF2. *KEAP1* mutations in lung cancer cells upregulate FSP1 expression, leading to resistance to ferroptosis and radiotherapy^181^. Additionally, NRF2 directly regulates the E3 ubiquitin ligases HERC2 and VAMP3. Therefore, *NRF2* knockout in ferroptosis-resistant ovarian cancer cells elevates apoferritin in the autophagosome and intracellular labile iron pool, ultimately leading to enhanced ferroptosis sensitivity^182^. These studies indicate that the NRF2 pathway plays a crucial role in the antiferroptosis mechanism.

Considering the role of both the HIF and NRF2 pathways in ferroptosis, associations between these signaling pathways and cancer are anticipated. However, comprehensive studies on this relationship are currently lacking. In noncancer cells, hypoxia-reperfusion induces ferroptosis, whereas NRF2 activation prevents ferroptotic cell death^71,183^. In gastric cancer cells, silencing of the transient receptor potential melastatin-2 (TRPM2) increases the levels of ROS, iron, and lipid peroxides, resulting in ferroptosis. In this study, TRPM-2 silencing-mediated ferroptosis was attributed to HIF-1α and NRF2 destabilization^184^. Elucidation of the roles of HIF and NRF2 in regulating ferroptosis will enable the development of novel cancer treatment strategies through the induction of ferroptosis.

### Concluding remarks

In the characteristic hypoxic and oxidative stress environment of tumors, increases in the levels of HIFs and NRF2 play a crucial role in tumor growth and progression. Elevations in the levels of these two transcription factors can occur not only due to environmental stimuli but also in response to changes in specific signaling pathways within the tumor. For instance, *KRAS* mutations increase NRF2 expression levels, promoting tumor growth^87^. When mutant *KRAS* was silenced in colorectal cancer cells, hypoxia-induced HIF-1α accumulation was suppressed^185^. Additionally, *PTEN* loss-mediated AKT activation and subsequent GSK-3β inhibition lead to an increase in NRF2^186^ and HIF-1α transcriptional activity^77^. Similarly, activation of human epidermal growth factor receptor 2 (HER2) results in an increase in HIF-1α expression levels via mTOR activation^77^. HER2 has also been reported to induce the stabilization of NRF2 through direct protein binding^187^. An oncometabolite, fumarate, simultaneously induces elevations in NRF2 levels through KEAP1 modification and activates HIF-1α through an increase in mitochondrial ROS^67,89^. These reports indicate a profound interconnection between tumor signaling pathways and the promotion of tumor growth and progression through increases in HIF and NRF2 levels, regardless of whether the tumor microenvironment is hypoxic or oxidative stress-enriched.

The control of the HIF and NRF2 signaling pathways in tumors has emerged as a critical issue. Current reports suggest that, during the interplay between these two transcription factors, NRF2 appears to be an upstream regulator of HIF. Inhibition of NRF2 in cancer cells has been reported to suppress HIF activity through multiple molecular events, including direct regulation of HIF-1α expression by NRF2, HIF-1α stabilization by the NRF2 target NQO1 and TRX1, and HO-1-driven CO and miRNA expression^58,59,61,65,66^. The relative dominance of NRF2 in the regulation of these two factors implies that HIFs evolved as a system responding to both hypoxic and oxidative stress, which is likely inevitable under low-oxygen conditions. In contrast, changes in NRF2 under hypoxic conditions vary in a context-dependent manner, indicating that hypoxia-associated oxidative stress does not necessarily accompany NRF2 activation. This relationship suggests that inhibiting NRF2 in tumors with both HIF and NRF2 upregulation could be a more efficient strategy considering the response of HIFs to hypoxia and oxidative stress. For instance, treatment with brusatol, an inhibitor of NRF2 protein synthesis, prevents hypoxia-induced HIF-1α accumulation and reduces glucose consumption in colorectal cancer cells^188^. Moreover, under sustained hypoxic conditions, brusatol inhibited the increase in HIF-2α and suppressed the development of hypoxia-induced CSC traits^66^.

On the other hand, reports indicate that a single substance can inhibit both HIFs and NRF2 through independent mechanisms. Triptolide, a diterpenoid isolated from *Tripterygium wilfordii*, has been shown to inhibit NRF2 and HIF-1α expression in myeloid leukemia cells^189^. In more detail, in doxorubicin-resistant or imatinib-resistant leukemic cells that highly express both NRF2 and HIF-1α, treatment with triptolide alone or in combination with the respective resistant drugs inhibited NRF2 and its target genes, as well as HIF-1α and its target genes. This inhibitory effect enhances the apoptotic ratio within cells, indicating that triptolide can restore drug sensitivity by suppressing the NRF2 and HIF-1α pathways. Furthermore, combined treatment with triptolide and idarubicin has synergistic effects on leukemia stem cells, enhancing cell apoptosis through the reduced expression of NRF2, HIF-1α, and their target genes^190^. Cardamonin, a natural chalcone from *Alpiniae katsumadai*, inhibits breast cancer cell growth by suppressing HIF-1α through blockade of the mTOR pathway and inducing metabolic reprogramming characterized by reduced glucose uptake and lactate transport^191^. Cardamonin also inhibited NRF2, NQO1, and HO-1 expression, thereby leading to increased ROS-induced apoptosis.

Overall, this review explored the intricate interplay between HIFs and NRF2, providing insights into the relevance of these interactions for the development of novel cancer treatment strategies. In particular, considering that both of these transcription factors also play critical roles in normal cell physiology, elucidating the common signaling pathways associated with their increase in tumors and revealing the molecular mechanisms underlying the correlation between these factors are expected to enable the development of the selective regulation of HIFs and NRF2 in cancers.
